# Seroprevalence of Brucella Infection and Its Determinants Among Pregnant Women Attending Antenatal Care in Burao City: At the Human Livestock Interface in Northeast Somaliland, Somalia

**DOI:** 10.1155/cjid/9922642

**Published:** 2025-02-25

**Authors:** Dek Kahin Yosef, Ahmed Saeed Ismail

**Affiliations:** ^1^School of Medical Laboratory Science, Department of Microbiology and Veterinary Public Health, Jigjiga University, Jigjiga, Ethiopia; ^2^University of Burao, Burao, Somalia; ^3^Faculty of Veterinary Medicine and Animal Production, University of Burao, Burao, Somalia

**Keywords:** brucellosis, infection, pregnancy, seroprevalence, Somaliland

## Abstract

**Background:** Brucellosis typically spreads from animals to humans through contact with infected animals or their byproducts. This zoonotic disease can have serious consequences and is often caused by contact with infected livestock or their products, such as contaminated dairy, posing significant risks during pregnancy. This study aimed to evaluate the prevalence of Brucella infection among pregnant women residing in the Burao City area of northeast Somaliland, in environments where human-animal interaction is a frequent occurrence. Therefore, it is important to identify the factors that contribute to its occurrence.

**Methods:** From January to June 2024, this cross-sectional study was conducted at five healthcare facilities that provide antenatal care. Pregnant women who attended these facilities were invited to participate in this study. A structured questionnaire was used to collect data on sociodemographic background, obstetric history, behaviors, and practices related to brucellosis. The presence of Brucella antibodies in the serum was detected using the Rose Bengal Plate Test (RBPT), and positive samples underwent further analysis with enzyme-linked immunosorbent assays (ELISA) to distinguish between IgG and IgM antibodies. Bivariate analysis was conducted to identify variables associated with Brucella seropositivity, whereas multivariable logistic regression was used to determine independent factors linked to Brucella seropositivity after adjusting for other variables.

**Results:** A total of 216 participants were included in the study. The overall prevalence of Brucella infection, determined using the RBPT, was 25.93% (56 out of 216). Among those who tested positive, 61.14% (34 out of 56) had IgG antibodies and 21.42% (12 out of 56) had IgM antibodies against Brucella, as confirmed by ELISA, and IgM ELISA testing revealed 5.6% of pregnant women had recent Brucella infections. Brucella seropositivity was found to be less likely for individuals who frequently interacted with manure, as indicated by an adjusted odds ratio of 0.052 and a 95% confidence interval of 0.016–0.169. Consumption of raw animal milk (AOR 4.84, 95% CI 2.24–10.42), and involvement in assisting animals during childbirth (AOR 4.26, 95% CI 1.065–17.0) significantly increased the risk of Brucella seropositivity.

**Conclusion:** Brucellosis poses a considerable public health threat to pregnant women residing in areas with frequent human-animal interactions. Factors such as the consumption of raw animal products, intimate contact with animals, and involvement in assisting with animal birth escalate this risk. These findings emphasize the importance of implementing strategies aimed at reducing exposure and enhancing the timely detection of brucellosis among pregnant women.

## 1. Background

Brucellosis is an often-under-recognized zoonotic illness that can be transmitted to humans through direct contact with infected animals, consumption of contaminated dairy products, or inhalation of airborne bacteria [1]. Domestic animals can carry various pathogens, making them reservoirs for potential diseases [1, 2]. Individuals who work closely with animals, such as veterinarians and livestock handlers, face a high risk of Brucella infection [1, 3]. While there is some evidence of brucellosis in sub-Saharan Africa, data from Somalia are limited and largely outdated, with most information predating the Civil War [4, 5]. Recent studies have suggested that sheep and goats have a brucellosis prevalence between 3% and 4.9%. Cattle seem to show rates from 5% to 9.5% when grazing freely in Somalia [6, 7].

For humans, getting infected with Brucella can be quite serious and is not just a minor health issue; it can affect entire communities and often requires long treatment times [8–10]. Sometimes, the symptoms look like other common illnesses, such as malaria or typhoid fever, which can lead to confusion and missed cases, especially where there are not good lab facilities available [11]. In humans, brucellosis can cause serious health issues, particularly in pregnant women who may experience complications such as miscarriage or preterm birth [1, 12, 13]. They may also deliver babies with low birth weights [13]. The impact of brucellosis is most severe among economically disadvantaged populations living near livestock with limited access to healthcare [1, 14]. Studies in Somalia revealed different rates among pregnant women, ranging from 1.30% to 5%, particularly in pastoral and agro-pastoral communities [7, 15].

In Burao City, Somaliland, data on Brucella infections among pregnant women are scarce, highlighting the need to determine the actual seroprevalence in this group. This study aimed to fill this gap by investigating Brucella infection prevalence among pregnant women at the human-livestock interface in Burao City.

## 2. Methods

### 2.1. Study Design and Area

This facility-based cross-sectional study was conducted in Burao District, Somaliland, between January and June 2024. The Burao District is characterized by camel, goat, and sheep rearing practices and has a population of approximately 126,000 individuals. The population of the Burao City District is approximately 12,600. Burao District has 11 public health facilities, comprising three health posts (hps), five maternal and child health centers (MCH/OPD), two MCH facilities, and one nonprivate hospital. Among the health facilities that provide antenatal services, two hospitals register between 25 and 40 new attendees per week. The study involved seven health facilities, including Burao General Hospital and other maternal and child health centers.

### 2.2. Study Population, Sampling Procedure, and Sample Size

This study enrolled pregnant women attending antenatal clinics at selected health facilities. Eligible participants were those who had resided in the study area for over 5 months and agreed to participate by signing a written informed consent form. The sample size of 216 pregnant women was calculated using the Kish Leslie formula, assuming a 16.9% seroprevalence of Brucella infection in Uganda [16]. Participants were enrolled consecutively until the required sample size was achieved.

### 2.3. Data Collection

Data were collected using a standardized questionnaire covering sociodemographic and obstetric characteristics, as well as potential risk factors for Brucella transmission [16]. This included interactions with animals and animal products, consumption of animal products, and individual-level exposure. To ensure the reliability of the questionnaire for assessing exposure to brucellosis risk factors, we developed the instrument based on validated tools from previous studies [1], conducted pilot testing, obtained expert reviews, provided thorough training to data collectors, and assessed internal consistency and test-retest reliability. Cronbach's alpha for multi-item scales was > 0.7, and kappa statistics for the test-retest reliability of key exposure variables was > 0.6, indicating acceptable reliability.

### 2.4. Study Variables

The dependent variable in this study was Brucella seropositivity, which was established using the results of the Rose Bengal Plate Test (RBPT). The independent variables were behaviors and practices that posed a potential risk of Brucella infection. To meet the criteria for regular contact with animal manure, participants were expected to have been unprotected from exposure to manure at least once a week for the past 5 months. Assistance in animal births was counted as contact with the placenta, with participants having to help animals give birth at least once in the past 5 months. Washing animals at home was counted if it was performed at least once a week for 3 months. Consuming foodstuffs such as fresh animal milk, raw animal blood, and raw meat was considered a preference if it was consumed at least once a week for the past 5 months.

### 2.5. Specimen Collection and Handling

Skilled healthcare professionals aseptically obtained 4 mL of venous blood by using a simple vacutainer system. The collected specimens were labeled with the participant's unique identification number. By spinning the samples at 3000 revolutions per minute for 5 minutes, serum was separated from the whole blood. The specimens were incubated at room temperature for 30 min and stored at 2°C–8°C until further processing.

### 2.6. Laboratory Test Procedure

#### 2.6.1. RBPT

Brucella serology was first recognized using rapid agglutination test [17]. However, this test does not differentiate between antibodies against various Brucella species. To administer the test, 50 μL of serum was carefully dispensed using a sterile micropipette onto the test plate, alongside an equal amount of RBPT antigen. The serum and antigen were then mixed using an applicator stick and gently rocked for 4 min before observation. A visible reaction was considered a positive outcome [18].

#### 2.6.2. Enzyme Linked Immunosorbent Essay (ELISA)

Positive RBPT samples were further tested using ELISA kits to detect IgG and IgM antibodies [1, 19]. Samples that were positive were stored at a temperature of minus 20 degrees Celsius before being transported to the reference laboratory at the Jigjiga University Molecular and Diagnostic Center and the Jigjiga Veterinary Regional Laboratory, Ethiopia. The purpose of this test was to detect the presence of Immunoglobulin M and G antibodies. Commercially available ELISA kits were used for this purpose. The assay was performed in accordance with the manufacturer's instructions. In this procedure, 100 mL of diluted serum samples and ready-to-use control solutions were added to the microtest wells that contained the antigen. The samples were then incubated at 37°C for 60 min. The first wash was performed after incubation. Following this, enzyme-conjugated anti-human IgM or IgG was added, and the mixture was incubated for 30 min at 37°C. After incubation, all wells were washed to remove any excess conjugate. The reaction was stopped by adding 100 mL of stopping solution. The enzyme reaction with the substrate produced a colored product, and the color intensity was proportional to the amount of specific antibody present. This product was measured using the photometric method.

### 2.7. Data Analysis

Categorical data were summarized using frequencies and percentages, whereas continuous data were summarized using medians. Pearson's chi-square test was used to evaluate differences between groups. Bivariate analysis was conducted to determine the variables associated with Brucella seropositivity, along with the crude odds ratio and 95% confidence interval [15]. Subsequently, multivariable logistic regression analysis was performed to examine the relationships between the outcome and independent variables.

To adjust for potential confounding variables in the multivariate logistic regression analysis, we used a stepwise approach. First, all variables that showed a significant association (*p* < 0.05) with Brucella seropositivity in the bivariate analysis were included in the initial model. We then used backward elimination, removing variables one at a time based on the highest *p* value until all remaining variables were significant at *p* < 0.05. Age and education level were retained in the model, regardless of significance, owing to their potential confounding effects. Multicollinearity was assessed using variance inflation factors (VIF), with a threshold of VIF > 5 indicating problematic collinearity. Interaction terms between key variables were tested and included if they were significant. The Hosmer–Lemeshow test was used to assess the goodness-of-fit of the final model. Adjusted odds ratios (AOR) with 95% confidence intervals were calculated to quantify the association between each variable and Brucella seropositivity, controlling for other factors in the model. All data analyses were conducted using STATA version 15, with the significance level set at 0.05.

## 3. Results

### 3.1. Sociodemographic Characteristics and Seropositivity of Brucella Infection

A total of 216 individuals were enrolled in the study, with an average age of 27 years and a range of 26–29 years. Almost all participants (93.5%) were married, 38.9% had completed secondary education, and 16.6% had pursued postgraduate studies. Among the 101 individuals who had previously been pregnant, 21 (21.9%) reported a history of spontaneous abortion. All participants underwent rapid RBPT to screen for Brucella antibodies, and 25.93% of the 216 tested were seropositive. Of the 34 seropositive individuals, 61.1% had IgG-specific antibodies and 12 (21.42%) had IgM-specific antibodies. Based on the presence of IgM antibodies, 5.6% (12/216) of the participants had a recent Brucella infection. The seropositivity rates for demographic factors such as age and education level, as well as obstetric characteristics such as the number of pregnancies and history of spontaneous abortion, were significantly associated (*p* < 0.05) with the exception of marital status, which was not statistically significant (Table 1)

### 3.2. Behavior and Practice Associated With Brucella Infection

Human: Several potential risk factors for brucellosis have been assessed and reported. Among individuals who were exposed to these factors, there were more cases of Brucella seropositivity. In bivariate analysis, regular contact with animal manure decreased the likelihood of Brucella seropositivity (cOR 0.053, 95% CI, 0.025–0.113). Additionally, assisting with animal parturition by handling placentas raised the odds of being seropositive (cOR 6.99, 95% CI 2.83, 17.23). Consumption of raw animal milk and raw meat, washing animals at home, slaughtering animals at home, and sharing water sources with animals were all significantly associated with Brucella seropositivity (*p* < 0.05). The odds of seropositivity among those who consumed raw animal milk and raw meat ranged from 4.84 to 0.10 (Table 2).

### 3.3. Multivariate Analysis of Factors Associated With Brucella Seropositive

Multivariable regression analysis was performed to determine the relationship between Brucella seropositivity and various independent variables. Bivariate analysis included variables with a significant association (*p* < 0.05). Multivariate logistic regression analysis revealed that regular contact with manure, preference for raw milk, and assistance from animals during parturition were significant risk factors for Brucella seropositivity (Table 3). However, slaughtering animals at home and consuming raw meat were not significantly associated with Brucella seropositivity after adjusting for other factors.

## 4. Discussion

This study found a high seroprevalence of Brucella antibodies (25.93%) among pregnant women attending antenatal care in Burao City, Somalia. Several key risk factors were identified through multivariate analysis, including consumption of raw milk (AOR: 4.84; 95% CI: 2.24, 10.42), contact with animal manure (AOR: 0.052; 95% CI: 0.016, 0.169), and assisting with animal births (AOR: 4.261; 95% CI: 1.065–17.0).

The observed seroprevalence is comparable to findings from other pastoralist communities, such as the 25% reported among pregnant women in Rwanda [20]. However, this is higher than the rates found in Uganda (17%) and Tanzania [21, 22]. The elevated seroprevalence in our study likely reflects the predominantly pastoralist population in Burao City, where close human-livestock interactions are common. Several factors may explain the differences in seroprevalence observed across these studies, including geographical variation, socioeconomic conditions, cultural practices, study population characteristics, timing, sampling methods, livestock density, public health interventions, diagnostic techniques, and zoonotic transmission patterns. These factors, individually or in combination, could account for the variations in the reported rates.

Consumption of raw milk has emerged as a significant risk factor, consistent with findings from Uganda, emphasizing the impact of food preferences on Brucella infection in pastoral communities [23]. The preference for raw milk consumption (reported by 57.41% of the participants) highlights the need for targeted education on safe food practices. Several factors contribute to the prevalence of raw milk consumption in Somaliland and Somalia, thereby increasing the risk of Brucella infection. Traditional dietary practices, limited access to pasteurization infrastructure, economic constraints, cultural beliefs, and a lack of awareness of the associated health risks all play a role. Additionally, challenges in maintaining proper cold chains, proximity to livestock in pastoral communities, limited food options, resistance to changing long-standing traditions, and insufficient regulatory oversight further contribute to the continued consumption of raw milk despite potential health hazards. Contrary to expectations, individuals in contact with animal manure had lower odds of testing positive than those without such contact. This inverse relationship may be influenced by various factors, including differences in exposure to specific pathogens and variations in hygiene practices among individuals. However, it is important to note that exposure to animal feces has been associated with health risks such as diarrhea and soil-transmitted helminth infections. This aligns with previous studies in Tanzania, Pakistan, Zambia, and Bangladesh, which identified constant exposure to animal feces as a risk factor [1, 12, 24, 25]. The high proportion of women reporting contact with animal manure (70.37%) underscores the importance of promoting hygienic practices when handling livestock. Regular contact with animal manure decreases the odds of seropositivity in Somaliland and Somalia because of various factors, including the prevalent pastoral lifestyle, limited sanitation infrastructure, water scarcity, traditional farming practices, lack of awareness about health risks, cultural practices involving close animal contact, economic constraints limiting access to protective equipment, and environmental conditions that may facilitate pathogen survival in animal waste.

Assisting with animal birth was associated with the highest odds of seropositivity. This aligns with previous studies in Punjab, Kenya, and Ethiopia [26–28]. This finding differs from that of a study in eastern Uganda, where assistance with animal births was not significantly associated with human brucellosis [29]. This discrepancy may reflect differences in local practices or the specific animal species involved. Given that 36.57% of the participants reported assisting with animal births, interventions to reduce exposure during these high-risk activities are crucial.

Although raw meat consumption (undercooked liver or meat) was significant in the bivariate analysis, it did not remain significant in the multivariate model. This contrasts with previous studies that identified raw meat as a risk factor [1, 30]. The lack of significance in our multivariate analysis may reflect the relative importance of different transmission routes in this population, or limitations in sample size may have limited our ability to detect smaller effect sizes in the multivariate analysis. Additionally, there may be confounding factors or interactions between variables that we were unable to fully account for in our model.

This study revealed a significant prevalence of recent Brucella infections among pregnant women, with 5.6% showing immunological evidence on IgM ELISA testing. This finding is inconsistent with those of some previous studies but differs from others, highlighting the variability in Brucella infection rates across different regions and populations. A study in Egypt reported a prevalence of Brucella infection of 12.2% among pregnant women using IgM ELISA [16]. Additionally, a study in Northern Tanzania found a higher prevalence of 23.5% using IgM ELISA [1]. The variation in prevalence rates across studies can be attributed to factors such as geographical differences, diverse animal husbandry practices, varying levels of public health awareness, and differences in diagnostic methods and study populations.

The finding that 5.6% of the participants had recent Brucella infections, as evidenced by IgM antibodies, has significant public health implications. This high rate of recent infections underscores the ongoing transmission of Brucella in the community and the urgent need for targeted interventions. This suggests that implementing routine Brucella screening as part of antenatal care could be beneficial in identifying and treating infections early, potentially reducing adverse pregnancy outcomes. Furthermore, this finding highlights the critical need for increased education on brucellosis transmission routes and prevention methods, particularly focusing on safe food practices and hygiene when handling livestock. Given the high risk associated with assisting animal births, occupational health measures such as providing protective equipment and training should be prioritized for those involved in these activities. The prevalence of recent infections also emphasizes the importance of adopting a One Health approach and integrating human, animal, and environmental health strategies to effectively control brucellosis in this region. Establishing ongoing surveillance systems for brucellosis in both human and animal populations is crucial for tracking the effectiveness of interventions and identifying emerging hotspots.

This study has several limitations that should be considered when interpreting the results. This cross-sectional design precludes the establishment of causal relationships between identified risk factors and Brucella infection. The sample size and facility-based recruitment may limit the generalizability of the results to all pregnant women in Burao City. Our sample was limited to women attending antenatal care at selected health facilities, which could potentially exclude women who do not access these services due to various barriers. Additionally, serological tests such as RBPT and ELISA in this setting suggest that efforts to expand laboratory capacity and train healthcare workers in these techniques could significantly enhance diagnostic capabilities and have inherent limitations, including potential false positives, cross-reactivity issues, and inability to distinguish between past and current infections.

Despite these limitations, our findings provide valuable insights into the prevalence and risk factors of Brucella infection among pregnant women in this region. These results have important implications for public health interventions and future research. They highlighted the need for targeted and culturally appropriate education programs that focus on safe food practices, hygiene when handling livestock, and protective measures during high-risk activities. Integrating Brucella screening and education into routine antenatal care can improve early detection and treatment. This study also demonstrated the feasibility of using serological tests in resource-limited settings. Future research, including longitudinal studies with larger sample sizes and community-based sampling approaches, is needed to better understand the dynamics of Brucella transmission in this population and to develop more effective prevention strategies.

## 5. Conclusion

This study revealed a high Brucella antibody prevalence (25.93%) among pregnant women in Burao City, Somalia, with key risk factors including raw milk consumption, contact with animal manure, and assisting animal births. The high rate of recent infections (5.6%) indicates ongoing transmission, emphasizing the need for intervention. These findings underscore the importance of targeted education on safe food practices and livestock handling, integrating Brucella screening into antenatal care, and adopting a one-health approach for control. To reduce infection rates, the focus should be on improving dietary habits, promoting pasteurization, enhancing hygiene when handling livestock, and minimizing exposure during high-risk activities. Implementing targeted screening programs, conducting public health education, improving livestock management, and strengthening food safety regulations are crucial steps. By addressing these aspects, we can work toward reducing the brucellosis burden in Burao City and similar resource-limited environments.

## Figures and Tables

**Table 1:** The descriptive features of the participants and Brucella seropositivity, as determined by the rapid RBPT.

Variables	Frequency	Seropositivity *N* (%)	*p* value
Overall seropositivity of brucellosis	216	56 (25.93)	

*Age group*			
18–21	20	4 (20)	≤ 0.006^∗^
22–25	54	5 (9.25)	
26–29	71	19 (16.7)	
30–33	42	16 (38.09)	
34–37	19	9 (47.3)	
> 37	10	5 (50)	

*Level of education*			
Primary	31	17 (55)	≤ 0.001
Secondary	84	23 (27.4)	
College or university	65	11 (17)	
Postgraduate	36	5 (13.9)	

*Marital status*			
Divorced	14	6 (42.9)	0.090
Married	202	50 (25.5)	

*Previous pregnancy*			
0	115	10 (8.7)	≤ 0.001
1	27	24 (89)	
2	31	14 (45.1)	
3+	43	8 (19)	

*Spontaneous abortion (n = 101)*			
No	80	11 (13.75)	≤ 0.001
Yes	21	7 (33.3)	

^∗^Indicates there is statistically significant association, *p* ≤ 0.05.

**Table 2:** Brucella seropositivity's associated factors in a bivariate analysi**s**.

Variable	Frequency	Seropositive *N* (%)	COR	95% CI	*p* value
*Raw milk*					
Yes	124	46 (37.09)	4.84	(2.28–10.25)	≤ 0.001
No	92	10 (10.9)	1	1	

*Frequent consumption raw milk*					
At least once per week	70	38 (54.2)	1	1	≤ 0.001
At least once per month	54	18 (33.3)	0.42	(0.20–0.88)	

*Sharing water source with animals*					
Yes	171	25 (14.6)	0.077	(0.036–0.165)	≤ 0.001
No	45	31 (68.88)	1	1	

*Raw meat*					
Yes	60	3 (5.0)	0.10	(0.03–0.34)	≤ 0.001
No	156	53 (33.9)	1	1	

*Washing animal at home*					
Yes	148	15 (10.13)	0.074	(0.036–0.153)	≤ 0.001
No	68	41 (60.29)	1	1	

*Slaughtering animal at home*					
Yes	76	8 (10.5)	0.23	(0.10–0.51)	≤ 0.001
No	140	48 (34.2)	1	1	

*Animal contact with manure*					
Yes	152	14 (9.21)	0.053	(0.025–0.113)	≤ 0.001
No	64	42 (66)	1	1	

*Assisted animal during parturition*					
Yes	79	6 (7.6)	6.99	(2.83–17.23)	≤ 0.001
No	137	50 (36.49)	1	1	

^∗^Indicates there is statistically significant association, *p* ≤ 0.05.

**Table 3:** Results of multivariate analysis examining the factors linked to RBPT seropositivity among participants.

Variable	Frequency	Seropositive *N* (%)	AOR	95% CI	*p* value
*Raw milk*					
Yes	124	46 (37.09)	4.84	(2.24, 10.42)	≤ 0.044^∗^
No	92	10 (10.9)	1	1	

*Animal contact with manure*					
Yes	152	14 (9.21)	0.052	(0.016, 0.169)	≤ 0.051^∗^
No	64	42 (66)	1	1	

*Assisted animal during parturition*					
Yes	79	6 (7.6)	4.26	(1.065, 17.0)	≤ 0.040^∗^
No	137	50 (36.49)	1	1	

^∗^Indicates there is statistically significant association *p* ≤ 0.05.

## Data Availability

Due to the privacy policy, the datasets are not publicly available. The corresponding author is happy to provide the necessary data upon request to support the study's findings.

## Nomenclature

ELISA: Enzyme-linked immunosorbent assay
IgG: Immunoglobin G
IgM: Immunoglobin G
RBPT: Rose Bengal Plate Test
